# Low Calorie Sweeteners Differ in Their Physiological Effects in Humans

**DOI:** 10.3390/nu11112717

**Published:** 2019-11-09

**Authors:** Stephanie R. Hunter, Evan J. Reister, Eunjin Cheon, Richard D. Mattes

**Affiliations:** Department of Nutrition Science, Purdue University, West Lafayette, IN 47907, USA; hunter99@purdue.edu (S.R.H.); ereister@purdue.edu (E.J.R.); cheone@purdue.edu (E.C.)

**Keywords:** low calorie sweeteners, body weight, appetite, energy intake, microbiota, sweet taste receptor, brain activation

## Abstract

Low calorie sweeteners (LCS) are prevalent in the food supply for their primary functional property of providing sweetness with little or no energy. Though tested for safety individually, there has been extremely limited work on the efficacy of each LCS. It is commonly assumed all LCS act similarly in their behavioral and physiological effects. However, each LCS has its own chemical structure that influences its metabolism, making each LCS unique in its potential effects on body weight, energy intake, and appetite. LCS may have different behavioral and physiological effects mediated at the sweet taste receptor, in brain activation, with gut hormones, at the microbiota and on appetitive responses. Further elucidation of the unique effects of the different commercially available LCS may hold important implications for recommendations about their use for different health outcomes.

## 1. Introduction 

Low calorie sweeteners (LCS) are prevalent in the food supply for their primary functional property of providing sweetness with little or no energy. This class of compounds is variously labeled, but it is argued that the term “low calorie sweetener” is preferred because it is an apt descriptor for communication in both the scientific and lay communities. Other terms are inaccurate or misleading: (A) “high intensity sweeteners” are not sweeter than sucrose, they only attain a given level of sweetness at a lower concentration; (B) “artificial sweeteners” is inaccurate because stevia is a natural product; (C) “non-caloric sweeteners” is invalid because aspartame yields energy (albeit little at concentrations commonly used) and (D) while “high potency sweeteners” is accurate, the term implies an inappropriate supra-physiological effect and/or a medicinal function. 

Arguably, the first LCS was lead acetate, used by the Romans. It was produced by heating grape juice in pots made of clay containing lead that leached from the vessel and bound to the acetate formed by the hydrolysis of acetic acid. When reduced in volume to 30% or 50% it was termed “sapa” or “defrutum,” respectively, and was added to foods and beverages [1]. Clearly, this was a potentially toxic food ingredient and many other sweeteners have been accidentally (e.g., saccharin, cyclamate, aspartame, sucralose) or purposefully identified and introduced into the food supply to serve a similar purpose with lower risk. The safety of each has been challenged and controversy continues [2,3], though extensive reviews by governmental agencies in numerous countries or regions have uniformly concluded they are safe when used as intended [4,5,6,7].

Though tested for safety individually, there has been extremely limited work on the efficacy of each LCS. That is, do they all elicit common behaviors such as reduced energy-yielding sweetener intake and physiological effects including modulated glycemia and lower body weight (BW)? It is assumed they act similarly. However, it is argued here that the rationale that they are all sweet and provide limited energy and thus evoke common responses is wanting. By analogy, both sodium chloride and potassium chloride are salty, yet the former purportedly raises blood pressure and the latter reduces it [8,9]. Tryptophan, strychnine and theophylline are all bitter but the former is an essential amino acid, strychnine is a toxin that inhibits respiration and theophylline is a treatment for asthma. Clearly, sensory property is not a reliable index of effect. This paper will present a viewpoint that the behavioral and physiological effects of each compound classified as a LCS should be examined individually.

## 2. Efficacy for Weight Loss

Both the food industry and consumers add LCS to their foods and beverages to maintain the palatability of products containing less sugar and energy. The goal is to reduce sugar intake, moderate swings of blood sugar and/or reduce or maintain BW. However, the evidence base supporting the use of LCS for these purposes is less than compelling, prompting relevant governmental and professional organizations to issue equivocal and inconsistent guidance on their use. The 2015 Dietary Guidelines for Americans report indicates that LCS may be a useful tool to lose weight in the short-term but their effectiveness as a long-term weight management strategy is questioned [10]. The American Heart Association holds a similar position, stating LCS beverages may be beneficial as replacements to reduce intake of sugar-sweetened beverages in the short-term for adults who are habitually high consumers of sugar-sweetened beverages [11]. However, it is still not prudent to recommend replacing sugar with LCS in children due to unknown effects of LCS use in the long-term. The position of the Academy of Nutrition and Dietetics states that consumers can safely enjoy a range of LCS when consumed within an eating plan that is guided by current federal nutrition recommendations, as well as individual health goals and personal preference [12]. The Canadian Diabetes Association reports that LCS may improve weight loss and weight control in obese persons if they are used as an adjunct to weight management programs [13].

The evidence pertaining to the use of LCS for weight management is notably inconsistent. Recent meta-analyses report that cohort studies examining LCS use yield findings of no association [14] or a positive association with body mass index (BMI) [15,16] or BW [16,17]. Meta-analyses of randomized controlled trials (RCT) indicate LCS use results in a lower [14,15] or no [16,17] association with BMI or BW (See Table 1). Much of the inconsistency stems from decisions made in selecting papers to include in the different meta-analyses (as will be critically reviewed below), but the possibility that inconsistencies are attributable to differences in the LCS used cannot be dismissed.

### 2.1. Evidence from Human Prospective Cohort Studies

In 2014, Miller and Perez [15] conducted a meta-analysis using PubMed and MeSH and relevant free text terms to search the literature through September 16, 2013 (with no lower date limit). The eligible studies included healthy adults or children with intake of at least one type of LCS and BW or composition outcomes for more than 6 months. Nine prospective cohort studies were included in the meta-analysis. Four of them were performed in children and adolescents and five in adults. The results were analyzed using random-effect modeling and showed a small but significant positive association between LCS use and BMI (Weighted group mean difference (WGMD): 0.03 kg/m^2^; 95% confidence interval (CI): 0.01, 0.06), but there was no significant association with BW (WGMD: 0.02 kg; 95% CI: −0.01, 0.06) or fat mass (WGMD: 0.035 kg; 95% CI: −0.026, 0.096).

The following year, Rogers et al. [14] reviewed the literature using MEDLINE and EMBASE databases via the OVID interface and Web of Science. MeSH terms and key words for sweetener types and outcomes (energy intake (EI) and anthropometric measures) were used to search the literature through 1 February, 2015 (with no lower date limit). Cohort studies were included if they had over 500 participants and more than 1 year of follow-up, which is a longer duration than Miller and Perez’s duration criteria. In addition, there was no limit on health status of the population, unlike the meta-analysis by Miller and Perez. The nine human trials with adults and three with children reported no significant change in BMI with LCS use compared to non-use (WGMD: −0.002 kg/m^2^; 95% CI: −0.009, 0.005). Some of the studies that were included in Miller and Perez’s meta-analysis were excluded in the Rogers et al. meta-analysis. For example, Johnson et al. [32] and Parker et al. [29] were excluded due to limited sample size. Newby et al. [24] was also excluded due to a follow-up period less than one year. The studies by Colditz et al. [28] and Schulze et al. [30] were excluded because a more recent paper by Pan et al. [21] analyzed the same cohort but with more detail. Nevertheless, the meta-analyses by Miller and Perez [15] and Rogers et al. [14] were more in agreement than disagreement, concluding LCS had minimal effects on BMI.

In 2017, Azad et al. [16] reported results from a meta-analysis that used MEDLINE, EMBASE and The Cochrane Central Register of Controlled Trials (CENTRAL) to search the LCS literature through 1 January, 2016 (with no lower date limit). Conference proceedings and search engines including OpenSIGLE and Google Scholar were also used. Cohort studies that were conducted longer than 6 months with both adults and children over 12 years of age were included, but studies with children were evaluated separately. As a result, eight cohort studies were screened for LCS effects on BMI and/or BW. The eight papers were evaluated for quality using the nine-point Newcastle Ottawa Scale and the studies with nine points were included in the meta-analysis. Due to the strict criteria, only two cohort studies on BMI and two on BW were eligible for inclusion. Notably, all of the papers except one contained in the prior meta-analyses by Miller and Perez [15] or Rogers et al. [14] were excluded in this later meta-analysis. Azad et al. [16] reported that both BMI (WGMD: 0.05 kg/m^2^; 95% CI: 0.03, 0.06) and BW (WGMD: 0.06 kg; 95% CI: 0.05, 0.07) were slightly positively associated with LCS use. However, one of the two BMI studies [22] assessed the role of diet soft drink consumption (among other behavioral factors) on the relationship between education level and BMI. The other study [27] assessed the role of soft drinks (regular and diet) and sports drinks on BMI in children 9–16 years old, but the kind or amount of LCS consumed was not quantified. Moreover, the Smith et al. [31] study included in the BW data was focused on evaluating different methodological approaches and lacked the appropriate controls to isolate LCS effects.

Most recently, in 2019 Toews et al. [17] reported results from a meta-analysis after a systematic literature review conducted through 25 May, 2017 (with no lower date limit) using MEDLINE, EMBASE, and CENTRAL databases. Eligibility criteria included a generally healthy population of adults (≥18 years) or children (<18 years), including those with overweight and obesity. All studies had to have more than seven days of intervention. To identify ongoing or completed but unpublished trials, the WHO International Clinical Trials Registry Platform (ICTRP) search portal and ClinicalTrials.gov were searched on 23 November, 2017. The relevant systemic literature reference list was also screened manually for potential citations. Only one prospective cohort study [28] was deemed eligible and the report did not include a meta-analysis for the lack of sufficient literature. The one eligible study showed that consuming up to 5.8 g saccharin per day was not associated with weight gain (WGMD: −0.09 95% CI:−0.13, 0.05) but was negatively associated with daily energy intake (WGMD: −1064.74 KJ, 95% CI: −1867.03, −262.44).

Overall, there was no or a small positive association between LCS consumption and BW in the prospective cohort studies. Importantly, there was limited overlap in included studies in the four meta-analyses despite being conducted within a tight time frame and sharing common outcomes. Additionally, substantive methodological questions have been raised about many of the papers, resulting in a lack of consistency in their inclusion and variable outcomes. Most of the cohort studies that were included in the four meta-analyses quantified LCS beverage intake frequency, but did not elucidate which LCS was consumed (see Table 1).

### 2.2. Evidence from Human Randomized Controlled Trials (RCTs)

In contrast to findings of observational studies, the intervention studies investigating the effect of LCS on BW predominantly report LCS usage being beneficial or neutral. In the previously mentioned meta-analysis by Miller and Perez [15], RCTs published prior to September 2013 were reviewed. Eligibility included an intervention duration of > 2 weeks and the control arm was sugar-sweetened beverages or supplements. Results showed significant benefits of LCS on BMI (WGMD: −0.24; 95% CI: −0.41, −0.07), BW (WGMD: −0.89 kg: 95% CI: −1.17, −0.43), fat mass (WGMD: −1.10 kg; 95% CI: −1.77, −0.44) and waist circumference (WGMD: −0.83 cm; 95% CI: −1.29, −0.37) compared with sugar consumption. The authors concluded that substituting LCS for the regular calorie versions of foods and beverages results in modest weight loss and LCS can be a useful tool to improve compliance during weight loss and maintenance. However, they did not analyze different LCS for individual effects on health outcomes.

Rogers et al. [14] also reviewed human RCT literature for an effect of LCS on BW. Studies were only included if there was an explicit instruction or requirement to consume LCS beverages or food as an alternative to or substitute for consumption of sugar-sweetened products or water. Studies that reported end points of EI and/or anthropometric measures were included. Participants were included if they were healthy independent of BW. Eligibility included a study duration of more than 4 weeks. Both short-term (less than 24 h) and longer term (more than 1 day) studies in humans found LCS use was associated with reduced EI compared with those consuming sugar-sweetened products (WGMD: −94 kcal, 95% CI: −122 to −66) and there was no difference in EI compared to those consuming water (WGMD: −2 kcal, 95% CI: −30 to −26). BW in studies longer than 4 weeks was also reduced after LCS consumption when compared to sugar-sweetened product (WGMD: −1.35 kg, 95% CI: −2.28 to −0.42) or water consumption (WGMD: −1.24 kg, 95% CI: −2.22 to −0.26). Although studies included in this meta-analysis used various combinations of LCS, Rogers et al. [14] did not conduct a sub-analysis for the individual LCS effects on health outcomes. The inclusion and exclusion criteria used by Rogers et al. [14] and Miller and Perez [15] differed. Miller and Perez [15] included studies lasting more than 2 weeks while Rogers et al. [14] only included studies lasting more than 4 weeks. Some of the studies included in Miller and Perez’s [15] meta-analysis were excluded in Rogers’s et al. [14] due to the form of LCS consumption (capsules) [55] or consumption of polyols (isomalt) [46]. Nevertheless, the findings from these two meta-analyses were comparable.

In the meta-analysis conducted by Azad et al. [16], cross-over studies were excluded, as were studies with intervention periods less than 6 months or those that included participants less than 12 years of age. This meta-analysis reported that LCS consumption was not correlated with BMI (WGMD: −0.37 kg/m^2^; 95% CI: −1.10, 0.36) or weight changes (WGMD: −0.17 kg; 95% CI: −0.54, 0.21). However, one of the included trials [34] was designed to measure the effects of stevioside (Stevia) on blood pressure rather than BMI. Another study [38] was a trial assessing the effects of diet beverage consumption compared to water consumption during a weight loss program, confounding the outcome assessment.

Most recently, the meta-analysis by Toews et al. [17] included both randomized and non-randomized controlled trials with a study duration of seven days or longer. Toews et al. [17] reported that LCS consumption has no significant association with BW (WGMD: 0.03 kg/m^2^; 95% CI: −0.03, 0.09) compared with the control group (either sugar/glucose/fructose/placebo). In this analysis, one study [53] had a duration of only 8 days and a small sample size (n = 9), likely precluding detection of any intervention effect. Another study [47] was designed to measure the effects of Reb A on blood pressure. Toews et al. [17] also did a subgroup analysis based on the duration of LCS intervention and EI. Short duration studies that lasted less than four weeks and used aspartame as the intervention did not show a significant association with energy intake (−598.94 KJ, 95% CI: −1445.24 to 247.36), while one 10-week study that used a combination of aspartame, cyclamate, acesulfame potassium (ace-K) and saccharin showed a negative correlation with energy intake (−2597.00 KJ, 95% CI: −3125.35 to −2068.65). Whether the discrepancy was due to study duration or type of LCS intervention cannot be dissociated.

Taken together, RCT results from four recent meta-analyses suggest that LCS consumption is not or slightly negatively associated with BW. However, different inclusion/exclusion criteria resulted in limited overlap in studies used in the four meta-analyses. Again, methodological limitations in the included studies leave open questions about the efficacy of LCS in weight management generally, and more specifically, the role of individual LCS.

The fundamental limitation of the meta-analyses is how the literature is reviewed and screened. Literature searching and data abstraction are two of the most important steps in conducting meta-analyses. Each meta-analysis had different inclusion and exclusion criteria and no meta-analysis had the same set of included literature. Moreover, studies of questionable relevance and methodology were included in the meta-analyses for the efficacy of LCS for weight loss. For instance, Azad et al. [16] included studies with a very small sample size and too short of a period to expect a weight change [53], as well as a study that aimed to see the effects of Reb A on blood pressure (not on BW or BMI) [47]. Studies that were excluded from the meta-analyses were mainly due to small sample size and limited study duration.

Future studies should attempt to elucidate the independent effects of different LCS on BMI, BW, EI and anthropometrical outcomes. A recent study [57] shows that different LCS (aspartame, Reb A, saccharin and sucralose) have different effects on BW over 12 weeks: BW was slightly reduced in subjects consuming sucralose, while other groups showed slight weight gain (saccharin) or no change (aspartame, stevia). These results indicate that different LCS might evoke different physiological effects and should therefore be analyzed separately.

## 3. Sweet Taste Receptor

### 3.1. Sweet Taste Receptors in the Oral Cavity

The prevailing view holds that taste is comprised of a limited number of primary qualities [58,59] and each provides unique information that aids identification and ingestion of needed nutrients and avoidance of unwholesome compounds [60]. These qualities are perceived through a transduction process where, initially, chemicals in foods interact with specific receptors primarily in the oral cavity. Here, the chemical energy of food is converted to an electrical signal that is conveyed to the central nervous system where it is decoded as a taste sensation. The sweet taste receptor, in particular, is credited with detecting carbohydrates that may be a useful source of energy. However, it can also detect sweetness in other classes of compounds such as some D-amino acids, and sweet-tasting proteins [61,62,63]. The receptor is a heterodimeric G protein coupled receptor (GPCR) formed by type 1, member 2 (T1R2) and type 1, member 3 (T1R3) protein subunits [64,65]. The T1R2 and T1R3 subunits share a common architecture, including a seven-helix transmembrane domain (TMD), a large amino terminal or venus flytrap domain (ATD/VFD) and an intermediate cysteine-rich domain (CRD) [66]. The T1R2 and T1R3 proteins are each able to bind selected sweeteners with distinct affinities [67]. Sucrose, glucose, sucralose and potentially saccharin and ace-K bind at the VFD of both T1R2 and T1R3 [67,68], whereas the VFD of T1R2 is thought to be the primary binding site of aspartame, neotame, and stevioside [69,70,71]. Additionally, cyclamate is thought to only bind to the TMD of T1R3 [70]. However, the literature on where exactly LCS bind on the sweet taste receptor is not straightforward. In humans, LCS achieve the sweetness of sucrose at concentrations that are 10^2^–10^5^ times lower than sucrose [72] probably due to stronger binding of LCS to the receptor [66]. For the present purpose, the key point is that different LCS bind to different sites on the sweet taste receptor and with different affinities.

This differential binding is associated with activation of distinct intracellular signaling. In rat taste receptor cells, sucrose increases the concentration of the secondary messenger cyclic adenosine monophosphate (cAMP) but saccharin has no effect [73]. Conversely, saccharin increases the secondary messenger inositol trisphosphate (IP_3_) in a dose-dependent manner, whereas sucrose only slightly raises IP_3_ [74]. Other studies of taste receptor cells have reported different patterns of changes in intracellular signals when comparing saccharin, sucralose and ace-K [75,76,77,78]. These LCS differentially impact intracellular cAMP and Ca^2+^ [75,76,77,78], indicating the activation of distinct transduction pathways based on molecular size and shape. One of the unique properties of the sweet taste receptor is its ability to generate multiple intracellular signals [75]. These various signals may cause different cellular outputs, but further research is needed to determine the downstream effects of LCS exposure.

The intracellular signals generated by LCS play a significant role in determining their sensory properties. Under specific conditions, ace-K is about 140× sweeter, aspartame 200× sweeter, saccharin 450× sweeter, sucralose 600× sweeter and neotame 11,000× sweeter than 5% sucrose [79]. These sweetness potencies correlate with detection thresholds, with the least amount required for neotame and the most for ace-K detection [79]. In terms of temporal profiles, the various LCS exhibit a range of onset, duration and decay times [80]. Besides differing in sweetness, most LCS demonstrate higher intensities of other taste and somatosensory notes such as bitterness and astringency when compared to caloric sugars [81]. Specifically, saccharin and ace-K, which are capable of binding to bitter taste receptors [82], leave an increasingly bitter taste as sweetness intensity increases [83]. Overall, LCS have unique binding capabilities, elicit different intracellular signaling patterns and manifest in distinct sensory properties. 

### 3.2. Sweet Taste Receptors outside the Oral Cavity

In addition to alerting the brain to a sweet stimulus, sweet taste receptor activation in the oral cavity also prepares the body to optimize food digestion and nutrient absorption through cephalic phase responses. The best studied cephalic phase response specifically relevant to sweeteners is the cephalic phase insulin response (CPIR). CPIR is a small, neurally-mediated release of insulin that occurs before nutrient absorption [84]. The energy-yielding sugars sucrose [85] and glucose [86,87] effectively stimulate the CPIR. Additionally, saccharin [85] and possibly sucralose [88] are effective CPIR stimulants. Interestingly, every sweet-tasting molecule demonstrated to produce CPIR is thought to be capable of binding the VFD of the T1R3 subunit [88]. Other LCS such as aspartame [89,90], stevioside and cyclamate [87] have not been reported to stimulate CPIR and bind elsewhere on the receptor. While it is possible that CPIR is mediated through the sweet taste receptor signaling pathway, recent evidence suggests that the adenosine triphosphate (ATP)-sensitive potassium channel (K_ATP_ channels) signaling pathway may be more instrumental to this process [91]. K_ATP_ channels have been found on T1R3-containing taste cells [92], but it is not known which (if any) LCS can activate this pathway. More research is needed on each LCS to determine whether or not they are capable of producing CPIR, but at present, evidence indicates this is limited to a sub-set of LCS. An effect of CPIR on carbohydrate metabolism has been documented [93,94,95], but the full health implications of the CPIR and other cephalic phase responses are not established, let alone the contributions of selected LCS.

In addition to their role in taste transduction in the oral cavity, sweet “taste” receptors have also been identified in various other organs, including the stomach, small intestine, large intestine, pancreas, adipose tissue, heart, brain, bladder and kidney [96]. These receptors function in a similar way in these extra-oral sites, i.e., activate intracellular signaling when a “sweet” ligand binds; however, by activating different types of cells, they evoke different physiological responses. Whereas specific functions of sweet taste receptors are mostly unknown in many areas of the body, their activation in the gastrointestinal tract and pancreas has been linked to increased incretin secretion and glucose-stimulated insulin expression [72,96], respectively, resulting in modulation of glucose homeostasis in animal models [96]. Studies of LCS in humans have provided inconsistent results [72]. Several experiments in healthy adults reported no effect of sucralose or aspartame consumption [97,98,99] or intragastric administration of sucralose, aspartame or ace-K [100,101,102] on intestinal glucose uptake or glucagon-like peptide-1 (GLP-1), peptide tyrosine tyrosine (PYY) or gastric inhibitory polypeptide (GIP) secretions. However, other work showed that sucralose consumption significantly increased GLP-1 secretion when compared to water consumption [103]. Additionally, studies have demonstrated that diet soda, the most common route of LCS consumption [104], is capable of increasing GLP-1 secretion in healthy adults and type I diabetics [105,106,107], suggesting that the taste or ingredients of diet soda are necessary to stimulate GLP-1 secretion [105]. It has been proposed that LCS disrupt glucose homeostasis by preparing the body for a carbohydrate load when none is forthcoming [108]. Taken together, individual LCS may differentially alter carbohydrate metabolism, but the evidence base is insufficient to draw firm conclusions.

## 4. Brain Activation

Sweet sensation activates areas of the brain involved in food memory and reward [109], but various sweet compounds differ in their specific effects. For example, the consumption of sucrose results in significantly greater global brain activation than both saccharin [110] and sucralose [111]. Specifically, sucrose elicits greater dopamine release than saccharin in the nucleus accumbens core [112] and greater activation than sucralose in the anterior insula, frontal operculum, striatum and anterior cingulate [111]. However, sucralose exposure leads to greater activation in the caudate [113] and stronger brain region connectivity than sucrose [111], indicating a more synchronized and efficient brain response. While the implication of this is uncertain, it is thought to be the result of the high affinity with which sucralose binds to the sweet taste receptor [66]. Another study reported that the taste of sucrose in caloric orangeade led to greater activation in the striatum than the taste of an LCS mixture (aspartame, ace-K, sodium cyclamate, and sodium saccharin) in non-caloric orangeade, but the taste of LCS elicited greater amygdala activation [114]. These findings indicate that the brain is capable of determining a difference between energy-yielding sugars and LCS, even if humans are not capable of consciously distinguishing between the two sensory stimuli [114]. 

Certain LCS activate similar areas of the brain as energy-yielding sugars, but this is not always the case. The consumption of glucose significantly decreases activation in the upper hypothalamus, whereas consumption of aspartame has no effect [89]. Oral exposure to glucose also increases activation in the anterior cingulate cortex and striatum, but no such response is observed after oral exposure to saccharin [115]. Lastly, while sucralose increases brain activation in many areas, sucrose activates several taste pathways (i.e., left ventral striatum, left dorsal caudate nucleus, bilateral midbrain and right thalamus) that sucralose does not activate [111]. Differences in brain activation between saccharin or sucralose and energy-yielding sugars are apparent, but direct comparisons between LCS are lacking. It is possible that LCS capable of binding bitter taste receptors, such as saccharin and ace-K [82], stimulate certain areas of the brain (i.e., amygdala) more than other LCS [114]. Additionally, the distinct binding sites and binding affinities of LCS may differentially influence brain activation.

A factor that may influence the response of the brain to both sugars and LCS is frequency of LCS consumption. One study noted a negative association between general LCS use and amygdala response to sucrose consumption, indicating a blunted activity response in the amygdala among frequent LCS consumers compared to its activity in less frequent LCS consumers [116]. Similarly, a study by Green and Murphy [117] reported a negative association between general LCS use (in the form of diet soda consumption) and caudate response to saccharin. The amygdala and caudate play an important role in modulating food motivation and reward, and it has been postulated that habitual LCS consumption uncouples sweet taste from energy intake [118]. An extension of this view holds that this uncoupling may induce incomplete energy compensation and ultimately increase food intake by activating food reward pathways [119]. Exposure to saccharin appears to disrupt food reward processes in rats [120,121], but this effect has yet to be shown in humans, as exposure to LCS beverages demonstrates no effect on reward value [122].

Currently, it is debated whether the attenuated response of the brain to LCS is beneficial or detrimental to energy intake and weight management. Sweet foods may be preferred and consumed because of increased activation of reward systems, or alternatively, preference and consumption may result from a lack of responsiveness of these systems [123,124]. In summary, evidence indicates differential brain responses between energy-yielding and LCS sweeteners. Less is known about differential responses between LCS, but it is possible that potential contrasts are a result of unique sensory properties among LCS.

## 5. Microbiota

There is suggestive evidence that the gut microbiota reportedly modulates appetite [125], food choice [126] and obesity [127]. Thus, changes in the composite, diversity and function of the microbiota leading to dysbiosis may compromise health. Obesity has been associated with an increased abundance of Firmicutes and a reduction in Bacteroidetes compared to microbial populations in lean individuals [128], an increased capacity to harvest dietary energy [129] and lower diversity in the gut microbiota [126]. At the same time, food choice, both acute and habitual [130], can modify the gut microbiota. Specifically, foods that are poorly digested in the stomach and small intestine that reach the colon, such as fiber rich foods, may become substrates for microbial fermentation [131]. These substrates influence the growth of select species of the gut microbiota and these species can generate signals that mimic and induce the release of satiety hormones from enteroendocrine cells and activate satiety centers in the brain via vagal afferents [132]. Therefore, dysbiosis in the gut microbiota could also negatively affect appetite. Due to the gut microbiota’s influence on appetite, food choice and BW, it is important to consider the role of LCS in the diet on the gut microbiota. 

The biological fate of different LCS determines their ability to influence the gut microbiota. Ace-K and aspartame are completely digested and their digestive products are absorbed in the small intestine and do not reach the colon [133]. Thus, they could not be expected to exert any direct effect on the microbiota. However, this does not rule out upstream influences through alteration of digestion, GI transit or absorptive processes. Such effects have been reported [134,135], but no mechanism has been identified and the findings have been challenged [136]. Saccharin, sucralose and steviol glycosides (stevia) reach the colon [133]. Steviol glycosides are further metabolized by gut bacteria, while saccharin and sucralose are excreted primarily intact in the feces [133]. Thus, steviol glycosides and possibly saccharin and sucralose have the potential to directly alter the gut microbiota. 

Animal models are frequently used to test the effects of LCS on the gut microbiota. Studies have reported that mice consuming 0.1 mg mL^−1^ of pure saccharin (equivalent to 5 mg/kg BW) [137] and 2–3 mg/kg BW stevia [138] induce dysbiosis in the gut microbiota, with mixed evidence for 1.1–15 mg/kg BW per day of sucralose [136,139]. Furthermore, the dysbiosis in the gut was associated with glucose intolerance in mice consuming 0.1 mg mL^−1^ of pure saccharin on a HFD [137], while the change in the gut microbiotadid not affect BW, food intake or insulin sensitivity in rats consuming 2–3 mg/kg BW of stevia [138]. However, food and water intake was only measured in a subset of mice consuming 0.1 mg mL^−1^ of pure saccharin, and may not be representative of the entire population [131,137]. Additionally, although glycemic response was significantly higher in mice consuming saccharin, about half of the mice still fell within a normal range, and it is not clear whether the fecal samples transplanted into germ free mice to determine dysbiosis were from mice with normal or high glycemic responses. Negative findings in a rat study on sucralose consumption may be due to non-standardized bacterial counts, no report of food consumption and an inability to rule out other ingredients in Splenda (i.e., maltodextrin) [139], thus questioning its validity. In addition, animal studies have made conclusions about all LCS despite only testing one specific LCS [137]. While animal studies report dysbiosis with different compositional changes to the gut microbiotaafter saccharin, sucralose and stevia consumption, they were not compared directly and these study limitations do not allow for clear evidence that any LCS has an effect, adverse or otherwise, on the gut microbiota in doses relevant to human consumption [131,140].

There are few studies that assess the effects of LCS consumption on the gut microbiota in humans. In a cross sectional study in 31 adults (seven were aspartame consumers (average ranged from 5.3 to 112 mg/day), seven were ace-k consumers (average ranged from 1.7 to 33.2 mg/day), while three consumed both LCS (amount not specified), determined by food records), there was no difference in bacterial abundance, but there were differences in bacterial diversity in fecal samples between aspartame consumers and non-consumers, ace-k consumers and non-consumers and LCS consumers and non-consumers [134]. As noted above, the rationale for this trial is unclear given aspartame and ace-K do not reach the colon and thus would not be expected to have a direct effect on the gut microbiota. Differences in bacterial diversity between LCS consumers and non-consumers in this study may stem from differences in habitual dietary intake [131]. A study evaluating long term consumption of LCS through food frequency questionnaires reported significant positive correlations between *Enterobacteriaceae* family, Deltaproteobacteria class and the Actinobacteria phylum in a randomly selected subgroup of LCS consumers independent of BMI [137]. LCS consumption was also associated with metabolic syndrome related parameters, including increased central obesity. These results suggest that the change in the gut microbiotaafter LCS consumption influences the change in metabolic syndrome-related parameters. However, the type of LCS and how much LCS was consumed were not reported, and whether other dietary factors influenced the gut microbiota and body composition is unknown [141]. Another small sample of seven healthy adults (who did not normally consume LCS) consumed the ADI of commercial saccharin (5 mg/kg BW) in three daily doses for 7 days [137]. Based on continuous glucose monitoring results, the seven participants were grouped into responders if they developed greater glycemic reactivity after 5–7 days of saccharin consumption and non-responders if there was no effect on glucose tolerance. Responders had pronounced compositional changes in their gut microbiota after 7 days of saccharin consumption, whereas non-responders had little change in composition. However, there was no control group, and whether the glucose intolerance was from saccharin is unknown. Diet composition was not controlled, and the gut microbiota composition between responders and non-responders was different before the intervention [141]. Stool samples from before and after 7 days of saccharin consumption from two responders and two non-responders were transferred to germ-free mice. The mice with stool from day 7 of saccharin consumption from responders developed glucose intolerance with the same microbiotacomposition as the donor, including increased *Bacteroides fragilis, Weissella cibaria*, and *Candidatus* Arthromitus. However, the mice transfected with stool from before and after 7 days of saccharin consumption from non-responders and mice transfected with stool from before saccharin consumption experienced neither glucose intolerance nor microbiota dysbiosis. The small sample size hampers interpretation of these findings.

While mice and human studies provide evidence that LCS consumption can alter the composition and diversity of the gut microbiota, how this influences appetite, energy intake and BW is unknown. Current studies largely focus on LCS effects on the gut microbiota in regards to glucose intolerance. There have been studies where LCS consumption led to weight gain despite no differences in energy intake [57,142]. The hypothesized mechanism is increased energy harvesting stemming from effects of LCS consumption on gut microbiota function, which may lead to increased BW in LCS consumers versus non-consumers. However, this is only speculative and requires verification. Which (if any) LCS increase energy harvest would also require verification. 

In addition to the different biological fates of the LCS, there are other factors that may influence differences in LCS effects on the gut microbiotathat make it difficult to interpret outcomes. Many animal studies use LCS in amounts in excess of the ADI [137,143,144,145], rendering extrapolations of findings to humans questionable. While animal studies typically use a controlled diet with LCS consumption, the human studies mentioned above did not control diet. Both food intake and diet composition can influence the gut microbiota; thus, whether differences are from LCS or the diet during the study period are not clear in human studies. Indeed, in the study by Suez et al. [137], there were compositional changes between responders and non-responders prior to consuming saccharin for 7 days, which may reflect different lifestyles between responders and non-responders. There are also differences in the gut microbiotaresponse to diet between individuals, making it difficult to generalize the effect of LCS specifically and especially in trials with small sample sizes [131]. Additionally, compared to fiber and resistant starch (consumed in grams), LCS are consumed at low amounts (mg) raising questions about their likely impact on the gut microbiota [146]. However, if they function more as signaling molecules rather than substrates, a role is feasible and there is preliminary evidence that LCS do have an impact on the gut microbiota. There is a need for more long term, randomized controlled trials in humans with controlled diets to assess the effect of LCS on the gut microbiotaand how they influence appetite, energy intake and BW. Due to their different biological fates, LCS must be evaluated individually to elucidate any effects on the gut microbiota.

## 6. Appetite

There have been many reviews assessing the role of LCS on appetite. Most conclude that LCS have little effect on appetite, especially when consumed in energy yielding foods [147]. However, concern persists that the sweetness of LCS stimulates hunger [148,149] and the desire for sweetness [147,150,151,152], thus compromising the intended effects of displacing energy. Most of this work has assessed appetitive responses acutely via preload design trials and findings have been extrapolated to longer-term implications for energy balance. Few trials have directly contrasted different LCS or examined the effects of chronic LCS consumption. An early trial indicated hunger ratings were higher after an aspartame preload compared to water alone whereas this did not hold for saccharin or ace-k preloads [148]. These findings were originally attributed to different biophysical properties of the sweeteners [153] or to palatability effects [154]. The validity of the finding is still open to question. Another trial contrasting effects of stevia, aspartame, and sucrose preloads on appetite did not replicate the appetite stimulating effect of aspartame [155]. However, aspartame increased hunger ratings at 30–60 min after the preload in [148], whereas the test meal was consumed 20 min after the preload in [155]; thus, the study designs may have accounted for differences in results. More recently, a 12-week randomized controlled trial contrasted the effects of sucrose, aspartame, saccharin, sucralose and reb-A on a number of health outcomes [57]. Adults with overweight or obesity consumed 1.25–1.75 L of a beverage sweetened with one of the sweeteners daily. Those consuming saccharin reported greater hunger ratings compared to all other treatment groups. Those in the saccharin group also had increased desire to eat and prospective consumption ratings at weeks 4, 8 and 12 compared to their baseline. This is despite no differences in energy intake between LCS groups. Thus, while the preponderance of evidence does not support an appetite augmenting property of LCS, there are short and long-term exposure trials revealing effects and ones that are different between LCS. These differences in LCS require further studies.

There is now increased understanding of potential differences in physiological responses to the different LCS and differences in their sensory properties are well-documented. Two other mechanisms warrant further exploration. The first implicates the effect of CPIR on appetite. The concept is that an increase in insulin release and subsequent decrease in blood glucose levels stimulate hunger [156], although there is evidence that indicates otherwise [147]. As stated previously, sucrose, glucose, saccharin and probably sucralose are effective CPIR stimulants, whereas aspartame, stevioside, ace-k and cyclamate have not been reported to stimulate CPIR. However, despite eliciting a CPIR, appetite ratings were not consistently correlated with insulin, and there was no effect on subjective feelings of appetite and energy intake at a meal 2 h after sham feeding sucralose [88]. Additionally, while CPIR was not measured specifically, saccharin and sucralose have been reported to have different effects on appetite and BW, which would not be attributed to CPIR since they both elicit a response [57]. While there are differences between LCS in their ability to stimulate a CPIR, few studies have assessed the role of LCS on the CPIR, and appetite. 

The second mechanism that warrants further exploration is the role of LCS on gut peptides. Due to their different biological fates, LCS can differentially stimulate gut peptides that play a role in appetite. GLP-1, cholecystokinin (CCK) and PYY are gut peptides that are associated with satiety and satiation. As mentioned previously, in vitro, animal and human studies provide conflicting evidence for the role of LCS in stimulating the release of gut peptides. Most in vitro studies indicate that LCS differentially stimulate the release of gut peptides, particularly GLP-1 and GIP [157,158,159,160], while animal studies indicate that different LCS do not stimulate the release of gut peptides [72,161]. Studies in human subjects indicate differences in gut peptide release depending on how LCS are consumed. Aspartame, ace-k or sucralose administered alone in human subjects do not have an effect on gut peptide release [100,102] or appetite [102]. However, when LCS are consumed with other LCS or other nutrients in foods and beverages or prior to an oral glucose tolerance test (OGTT), gut peptide release is augmented. Notably, sucralose may enhance the release of GLP-1 [103,107], but not GIP or PYY [107], when consumed with other LCS, in diet soda, or before an OGTT, but aspartame did not have an effect on gut peptide release [103]. However, this effect was not consistent. To our knowledge, only one study has compared the effect of different LCS consumption on gut peptide release and appetite [101]. In this placebo-controlled, double-blind, six-way, cross-over trial, 12 healthy subjects received an intragastric infusion of equisweet glucose, fructose, aspartame, ace-K or sucralose dissolved in 250 ml of water or water only as a control, and venous blood samples were collected for three hours after the infusion. While glucose stimulated GLP-1 and PYY secretion, and glucose and fructose increased satiety and fullness insignificantly compared to water, there were no effects of aspartame, ace-K or sucralose on gut peptide secretion or appetite. Inconsistent results may be due to differences in test solutions. For example, diet cola sweetened with sucralose and ace-K augmented GLP-1 release in response to an OGTT with no change in appetite compared to sucralose and ace-K in water [105]. Thus, other ingredients in the diet soda may be responsible for differences in GLP-1 rather than the LCS [107]), or stimulation of oral sweet taste receptors by LCS are required for intestinal GLP-1 release [107]. Thus, while LCS consumed alone may not stimulate the release of gut hormones, LCS, particularly sucralose, consumed with other nutrients may stimulate the release of GLP-1, but this finding is not robust. In addition to the controversial role of gut peptides at physiological levels on appetite, there is not enough evidence to suggest that gut peptide release is or is not a mechanism by which different LCS influence appetite.

## 7. Conclusions

It is often assumed that all LCS elicit common sensory responses, physiological processes and behaviors. Thus, they are often grouped as an entity in research studies and recommendations. However, each LCS has its own chemical structure that influences its metabolism and other downstream effects, making each LCS unique in its potential influence on appetite, food intake and BMI. Observational studies and RCTs report inconsistent evidence of LCS effects on these outcomes. This may stem from differential effects of the various LCS, but more studies are needed to elucidate this. LCS mechanisms for reducing BW may not be limited to energy dilution. LCS may have different behavioral and physiological effects mediated at the sweet taste receptor, with gut hormones, through brain activation, on the microbiota, and on appetitive sensations. Specifically, more research is needed to determine the extent to which individual LCS influence CPIR, gut hormone release and subsequent carbohydrate metabolism, neural responses and food reward, the composition and diversity of the gut microbiota and long-term appetitive responses. Further elucidation of the unique effects of the different commercially available LCS may hold important implications for recommendations about their use for different health outcomes.

## Figures and Tables

**Table 1:** Screening criteria for recent meta-analyses (I = included paper).

**SCREENING CRITERIA**							
**Meta-Analysis Papers**		**Miller, 2014** [15]	**Rogers, 2016** [14]	**Azad, 2017** [16]		**Toews, 2019**	
**Inclusion Criteria**		(1) Study population was generally healthy. (2) Studies in which dose or intake data for at least one LCS/polyol was provided. (3) The effect of LCS, compared with the control arm, could be examined independently of other intervention components. (4) Outcome data for at least one measure of body weight or composition were available.	(1) Observational Studies Studies of over 500 participants that reported on prospective analyses with more than 1 year of follow-up Where there were several reports of the same study, either the most detailed report of the results or the report with the longest follow-up were included. (2) RCTs Studies in which there was an explicit instruction or requirement to consume LCS foods or beverages as an alternative to or substitute for consumption of sugar-sweetened products, water or habitual diet (the comparison treatment). Studies in which participants had ad libitum access to (other) dietary energy sources. Studies with reported end points that included EI and/or anthropometric measures. Studies had to have a parallel or balanced-ordered cross-over design with healthy participants (regardless of BW) who were either uninformed or correctly informed of the manipulation (not deceived).	(1) Prospective, randomized, controlled trials of LCS interventions, or prospective observational cohort studies reporting LCS exposure. (2) Studies with LCS exposure reported in adolescents (over 12 years of age) or adults. (3) Minimum study duration of 6 months.		(1) Studies with a generally healthy population of adults (≥18 years) or children (<18 years), including those with overweight or obesity. Studies with overweight or obese individuals who were specifically trying to lose weight were analyzed separately. (2) Studies that applied concomitant interventions were included as long as the interventions were similar and equally balanced between the intervention and comparator groups to establish fair comparisons. (3) Studies that reported LCS use within the ADI as established by JECFA, EFSA, or FDA, or did not report information on dose. (4) Parallel groups or crossover (quasi-) RCTs and cluster randomized trials. (5) Non-randomized controlled trials, as well as prospective and retrospective cohort studies, case-control studies and cross sectional studies were included, but analyzed separately. (6) Unpublished and ongoing studies were included.	
**Exclusion Criteria**		Hospitalized or acutely ill populations	(1) Most sugar alcohols (2) Observational studies Studies that reported on LCS consumption and metabolic syndrome but did not report separate associations for an anthropometric component of metabolic syndrome. Papers published only as an abstract	(1) Not original research (i.e., reviews, commentaries) (2) Non-human studies (3) No outcomes of importance to the review were reported or available via contact with study authors (4) Observational Studies Cross sectional or retrospective design studies Analysis of LCS effects was not fully prospective (associations presented for change in LCS intake but not for baseline intakes) LCS associations with outcomes of interest were not adjusted for confounders/covariates. (5) Trials Quasi-randomized, cross-over or cluster randomized design studies LCS effects could not be examined independently of other intervention components		(1) Use of LCS exceeded the ADI (2) Diseased populations, in vitro and animal studies (3) Studies with pregnant women (4) Studies that did not specify the type of sweetener	
**Database**		PubMed Search ending on 16 September 2013.	OVID and web of science (MEDLINE/EMBASE) Reference lists of review papers Search ending on 1 February 2015.	OVID (MEDLINE) Review reference list Gray literature from OpenSIGLE and Google Scholar Search ending on 1 January 2016.		*Published Papers* *(1946-25, May 2017)* Medline Medline in Process Medline Daily Update Embase The Cochrane Central Register of Controlled Trials *Unpublished Papers* WHO International Clinical Trials Registry Platform ClinicalTrials.gov Search ending on 23 November 2017	
**Sweeteners**		LCS, polyols	LCS	LCS, xylitol		LCS approved by FDA, as well as brazzein and thaumatin	
**Controls**		Sugar	Water or sugar (analyzed separately)	Mixed control (water, placebo, avoidance)		Sugar/placebo/any other alternative intervention	
**Study Duration**	**Cohort**	>6 months	>1 year	>6 months		>7 days	
	**RCT**	>2 weeks	>4 weeks	>6 months		>7 days	
**RESULTS**							
**I. COHORT STUDIES**							
**1. BMI**		**Included Papers**	**LCS**	**Miller**	**Rogers**	**Azad**	**Toews**
**Adults**		Fowler et al., 2008 [18]	LCS Beverage	I	I		
		Chen et al., 2009 [19]	LCS Beverage		I		
		Vanselow et al., 2009 [20]	LCS Beverage		I		
		Pan et al., 2013 [21]	LCS Beverage		I		
		Gearon et al., 2014 [22]	LCS Beverage			I	
**Children**		Berkey et al., 2004 [23]	LCS Beverage	I	I		
		Newby et al., 2004 [24]	LCS Beverage	I			
		Striegel-Moore et al., 2006 [25]	LCS Beverage		I		
		Laska et al., 2012 [26]	LCS Beverage	I	I		
		Field et al., 2014 [27]	LCS Beverages			I	
**Combined** **Results**		Mean Difference (kg/m^2^)		0.03	−0.002/year	0.05	No Results
		Confidence Interval		(0.01, 0.06)	(−0.009, 0.005)	(0.03, 0.06)	No Results
		Conclusion		Positive	No Significance	Positive	No Results
**2. Body Weight**		**Included Papers**	**LCS**	**Miller**	**Rogers**	**Azad**	**Toews**
**Adults**		Colditz et al., 1990 [28]	Saccharin	I			I
		Parker et al., 1997 [29]	Saccharin	I		I	
		Schulze et al., 2004 [30]	LCS Beverage	I			
		Smith et al., 2015 [31]	LCS Beverage			I	
**Children**		Newby et al., 2004 [24]	LCS Beverage	I			
**Combined** **Results**		Mean Difference (kg)		0.02	No Results	0.06	No Results
		Confidence Interval		(−0.01, 0.06)	No Results	(0.05, 0.07)	No Results
		Conclusion		No Significance	No Results	Positive	No Results
**3. Fat Mass**		Included Papers	LCS	Miller	Rogers	Azad	Toews
**Children**		Laska et al., 2012 [26]	LCS Beverage	I			
		Johnson et al., 2007 [32]	LCS Beverage	I			
**Combined** **Results**		Mean Difference (kg)		0.035	No Results	No Results	No Results
		Confidence Interval		(−0.026, 0.096)	No Results	No Results	No Results
		Conclusion		No Significance	No Results	No Results	No Results
**II. RANDOMIZED CONTROLLED TRIALS**							
**1. BMI**		**Included Papers**	**LCS**	**Miller**	**Rogers**	**Azad**	**Toews**
**Adults**		Kanders et al., 1988 [33]	Aspartame	I			
		Hsieh et al., 2003 [34]	Stevioside powder			I	
		Ferri et al., 2006 [35]	Stevioside (3.75 mg/kg/day-7 weeks) (7.5 mg/kg/day-11 weeks) (15 mg/kg/day-6 weeks)			I	
		Reid et al., 2007 [36]	Aspartame	I	I		I
		Njike et al., 2011 [37]	Ace-K/aspartame	I			
		Madjd et al., 2015 [38]	Not specified			I	
**Children**		Ebbeling et al., 2006 [39]	21.7 oz/day of beverage, LCS not specified	I			
		Ebbeling et al., 2012 [40]	10.08 oz/day of beverage, LCS not specified	I			
**Combined** **Results**		Mean Difference (kg/m^2^)		−0.24	No Results	−0.37	No Results
		Confidence Interval		(−0.41, −0.07)	No Results	(−1.10, 0.36)	No Results
		Conclusion		Negative	No Results	No Significance	No Results
**2. Body Weight**		**Included Papers**	**LCS**	**Miller**	**Rogers**	**Azad**	**Toews**
**Adults**		Blackburn et al., 1997 [41]	Aspartame	I	I	I	
		Gatenby et al., 1997 [42]	Reduced-sugar foods	I			
		Kanders et al., 1988 [33]	Aspartame	I			
		Tordoff et al., 1990 [43]	Aspartame-sweetened soda	I			
		Raben et al., 2001 [44]	Mixture of LCS 54% aspartame +22% Ace-K +23% cyclamate +1% saccharin				I
		Raben et al., 2002 [45]	Mixture of LCS 54% aspartame +22% Ace-K +23% cyclamate +1% saccharin	I	I		
		Gostner et al., 2005 [46]	Polyolsomalt (Palatinit)	I			
		Reid et al., 2007 [36]	Aspartame		I		
		Maki et al., 2008 [47]	Aspartame				I
		Njike et al., 2011 [37]	Ace-K- and aspartame- sweetened hot cocoa	I	I		
		Reid et al., 2010 [48]	Aspartame	I	I		
		Maersk et al., 2012 [49]	Aspartame	I	I	I	I
		Tate et al., 2012 [50]	Diet beverage, LCS not specified	I	I	I	
		Reid et al., 2014 [51]	Aspartame				I
		Peters et al., 2014 [52]	Not specified		I		
		Madjd et al., 2015 [38]	250 ml/day of diet beverage, LCS not specified			I	
		Kuzma et al., 2015 [53]	Fructose-, glucose-, or aspartame-sweetened beverage				I
		Peters et al., 2016 [54]	Not specified			I	
**Children**		Knopp et al., 1976 [55]	Aspartame capsule	I			
		de Ruyter et al., 2012 [56]	34 mg sucralose +12 mg Ace-K	I	I		
		Ebbeling et al., 2006 [39]	21.7 oz/day of beverage, LCS not specified	I			
**Combined** **Results**		Mean Difference (kg)		−0.8 ^1^	−1.35 ^1^	−0.17 ^2^	Adult: −1.29 ^1^ Children: −0.6 ^1^
					1.24 ^3^		
		Confidence Interval		(−1.17, −0.43) ^1^	(−2.28, −0.42) ^1^ (−2.22, −0.26) ^3^	(−0.54, 0.21) ^2^	Adult: (−2.80, 0.21) ^1^ Children: (−1.33, 0.14) ^1^
		Conclusion		Negative	Negative ^1,3^	No Significance	No Significance
**3. Fat Mass**		**Included Papers**	**LCS**	**Miller**	**Rogers**	**Azad**	**Toews**
**Adults**		Kanders et al., 1988 [33]	Aspartame	I			
		Maersk et al., 2012 [49]	Aspartame	I			
		Raben et al., 2002 [45]	Mixture of LCS 54% aspartame +22% Ace-K +23% cyclamate +1% saccharin	I			
**Children**		Ebbeling et al., 2006 [39]	21.7 oz/day of beverage, LCS not specified	I			
		de Ruyter et al., 2012 [56]	34 mg sucralose +12 mg Ace-K	I			
**Combined** **Results**		Mean Difference (kg)		−1.1	No Results	No Results	No Results
		Confidence Interval		(−1.77, −0.44)	No Results	No Results	No Results
		Conclusion		Negative	No Results	No Results	No Results
**4. Waist Circumference**		**Included Papers**	**LCS**	**Miller**	**Rogers**	**Azad**	**Toews**
**Adults**		Njike et al., 2011 [37]	Ace-K and aspartame- sweetened hot cocoa	I			
		Tate et al., 2012 [50]	Diet beverage, LCS not specified	I			
**Children**		Ebbeling et al., 2006 [39]	21.7 oz/day of beverage, LCS not specified	I			
		de Ruyter et al., 2012 [56]	34 mg sucralose +12 mg Ace-K	I			
**Combined** **Results**		Mean Difference (cm)		−0.83	No Results	No Results	No Results
		Confidence Interval		(−1.29, −0.37)	No Results	No Results	No Results
		Conclusion		Negative	No Results	No Results	No Results

^1^ Compared to sucrose consumption group; ^2^ Compared to mixed control (sucrose, water, or placebo); ^3^ Compared to water consumption group. Abbreviations: LCS: low-calorie sweeteners, RCTs: randomized controlled trials, ADI: acceptable daily intake, JECFA: Joint FAO/WHO Expert Committee on Food Additives, EFSA: European Food Safety Authority, FDA: United States Food and Drug Administration, EI: energy intake, BW: body weight, WHO: World Health Organization, BMI: body mass index, Ace-K: acesulfame potassium.

## References

[1] Grocock C., Grainger S. (2006). Apicius: A Critical Edition with an Introduction and English Translation.

[2] Schiffman S.S., Rother K.I. (2013). Sucralose, A Synthetic Organochlorine Sweetener: Overview of Biological Issues. J. Toxicol. Environ. Health Part B.

[3] Swithers S.E. (2013). Artificial sweeteners produce the counterintuitive effect of inducing metabolic derangements. Trends Endocrinol. Metab..

[4] Panel on Food Additives and Nutrient Sources Added to Foodmill (2013). Scientific Opinion on the re-evaluation of aspartame (E 951) as a food additive. EFSA J..

[5] Serra-Majem L., Raposo A., Aranceta-Bartrina J., Varela-Moreiras G., Logue C., Laviada H., Socolovsky S., Perez-Rodrigo C., Aldrete-Velasco J.A., Meneses Sierra E. (2018). Libero(-)American Consensus on Low- and No-Calorie Sweeteners: Safety, Nutritional Aspects and Benefits in Food and Beverages. Nutrients.

[6] Roberts A. (2016). The safety and regulatory process for low calorie sweeteners in the United States. Physiol. Behav..

[7] Kroger M., Meister K., Kava R. (2006). Low-calorie Sweeteners and Other Sugar Substitutes: A Review of the Safety Issues. Compr. Rev. Food Sci. Food Saf..

[8] He Feng J., Markandu Nirmala D., Coltart R., Barron J., MacGregor Graham A. (2005). Effect of Short-Term Supplementation of Potassium Chloride and Potassium Citrate on Blood Pressure in Hypertensives. Hypertension.

[9] Rust P., Ekmekcioglu C. (2017). Impact of Salt Intake on the Pathogenesis and Treatment of Hypertension. Adv. Exp. Med. Biol..

[10] USDA Scientific Report of the 2015 Dietary Guidelines Advisory Committee Report. https://health.gov/dietaryguidelines/2015-scientific-report/pdfs/scientific-report-of-the-2015-dietary-guidelines-advisory-committee.pdf.

[11] Johnson R.K., Lichtenstein A.H., Anderson C.A.M., Carson J.A., Despres J.-P., Hu F.B., Kris-Etherton P.M., Otten J.J., Towfighi A., Wylie-Rosett J. (2018). Low-Calorie Sweetened Beverages and Cardiometabolic Health: A Science Advisory from the American Heart Association. Circulation.

[12] Fitch C., Kathryn K.S., Academy of Nutrition and Dietetics (2012). Position of the Academy of Nutrition and Dietetics: Use of Nutritive and Nonnutritive Sweeteners. J. Acad. Nutr. Diet..

[13] Gougeon R., Spidel M., Lee K., Field C.J. (2004). The Current Canadian Diabetes Association Clinical Practice Guidelines for the Prevention and Management of Diabetes in Canada Canadian Diabetes Association National Nutrition Committee Technical Review: Non-Nutritive Intense Sweeteners in Diabetes Management. Can. J. Diabetes.

[14] Rogers P.J., Hogenkamp P.S., de Graaf C., Higgs S., Lluch A., Ness A.R., Penfold C., Perry R., Putz P., Yeomans M.R. (2016). Does Low-Energy Sweetener Consumption Affect Energy Intake and Body Weight? A Systematic Review, Including Meta-Analyses, of the Evidence from Human and Animal Studies. Int. J. Obes..

[15] Miller P.E., Perez V. (2014). Low-Calorie Sweeteners and Body Weight and Composition: A Meta-Analysis of Randomized Controlled Trials and Prospective Cohort Studies. Am. J. Clin. Nutr..

[16] Azad M.B., Abou-Setta A.M., Chauhan B.F., Rabbani R., Lys J., Copstein L., Mann A., Jeyaraman M.M., Reid A.E., Fiander M. (2017). Nonnutritive Sweeteners and Cardiometabolic Health: A Systematic Review and Meta-Analysis of Randomized Controlled Trials and Prospective Cohort Studies. CMAJ.

[17] Toews I., Lohner S., de Gaudry D.K., Sommer H., Meerpohl J.J. (2019). Association between Intake of Non-Sugar Sweeteners and Health Outcomes: Systematic Review and Meta-Analyses of Randomised and Non-Randomised Controlled Trials and Observational Studies. BMJ.

[18] Fowler S.P., Williams K., Resendez R.G., Hunt K.J., Hazuda H.P., Stern M.P. (2008). Fueling the Obesity Epidemic? Artificially Sweetened Beverage Use and Long-Term Weight Gain. Obesity.

[19] Holt G.M., Owen L.J., Till S., Cheng Y., Grant V.A., Harden C.J., Corfe B.M. (2017). Systematic Literature Review Shows That Appetite Rating Does Not Predict Energy Intake. Crit. Rev. Food Sci. Nutr..

[20] Vanselow M.S., Pereira M.A., Neumark-Sztainer D., Raatz S.K. (2009). Adolescent Beverage Habits and Changes in Weight over Time: Findings from Project EAT. Am. J. Clin. Nutr..

[21] Pan A., Malik V.S., Hao T., Willett W.C., Mozaffarian D., Hu F.B. (2013). Changes in Water and Beverage Intake and Long-Term Weight Changes: Results from Three Prospective Cohort Studies. Int. J. Obes..

[22] Gearon E., Peeters A., Hodge A., Backholer K. (2014). The Role of Dietary and Physical Activity Behaviours in Educational Differences in Weight Gain among Australian Adults—The Melbourne Collaborative Cohort Study. Obes. Res. Clin. Pract..

[23] Berkey C.S., Rockett H.R., Field A.E., Gillman M.W., Colditz G.A. (2004). Sugar-added beverages and adolescent weight change. Obes. Res..

[24] Newby P.K., Peterson K.E., Berkey C.S., Leppert J., Willett W.C., Colditz G.A. (2004). Beverage Consumption Is Not Associated with Changes in Weight and Bodys Mass Index among Low-Income Preschool Children in North Dakota. J. Am. Diet. Assoc..

[25] Striegel-Moore R.H., Thompson D., Affenito S.G., Franko D.L., Obarzanek E., Barton B.A., Schreiber G.B., Daniels S.R., Schmidt M., Crawford P.B. (2006). Correlates of Beverage Intake in Adolescent Girls: The National Heart, Lung, and Blood Institute Growth and Health Study. J. Pediatrics.

[26] Laska M.N., Murray D.M., Lytle L.A., Harnack L.J. (2012). Longitudinal Associations between Key Dietary Behaviors and Weight Gain over Time: Transitions through the Adolescent Years. Obesity.

[27] Field A.E., Sonneville K.R., Falbe J., Flint A., Haines J., Rosner B., Camargo C.A. (2014). Association of Sports Drinks with Weight Gain among Adolescents and Young Adults. Obesity.

[28] Colditz G.A., Willett W.C., Stampfer M.J., London S.J., Segal M.R., Speizer F.E. (1990). Patterns of Weight Change and Their Relation to Diet in a Cohort of Healthy Women. Am. J. Clin. Nutr..

[29] Parker D.R., Gonzalez S., Derby C.A., Gans K.M., Lasater T.M., Carleton R.A. (1997). Dietary Factors in Relation to Weight Change among Men and Women from Two Southeastern New England Communities. Int. J. Obes..

[30] Schulze M.B., Manson J.A.E., Ludwig D.S., Colditz G.A., Stampfer M.J., Willett W.C., Hu F.B. (2004). Sugar-Sweetened Beverages, Weight Gain, and Incidence of Type 2 Diabetes in Young and Middle-Aged Women. J. Am. Med. Assoc..

[31] Smith J.D., Hou T., Hu F.B., Rimm E.B., Spiegelman D., Willett W.C., Mozaffarian D. (2015). A Comparison of Different Methods for Evaluating Diet, Physical Activity, and Long-Term Weight Gain in 3 Prospective Cohort Studies. J. Nutr..

[32] Johnson L., Mander A.P., Jones L.R., Emmett P.M., Jebb S.A. (2007). Is Sugar-Sweetened Beverage Consumption Associated with Increased Fatness in Children?. Nutrition.

[33] Kanders B.S., Lavin P.T., Kowalchuk M.B., Greenberg I., Blackburn G.L. (1988). An Evaluation of the Effect of Aspartame on Weight Loss. Appetite.

[34] Hsieh M.-H., Chan P., Sue Y.-M., Liu J.-C., Liang T.H., Huang T.-Y., Tomlinson B., Chow M.S.S., Kao P.-F., Chen Y.-J. (2003). Efficacy and Tolerability of Oral Stevioside in Patients with Mild Essential Hypertension: A Two-Year, Randomized, Placebo-Controlled Study. Clin. Ther..

[35] Ferri L.A., Alves-Do-Prado W., Yamada S.S., Gazola S., Batista M.R., Bazotte R.B. (2006). Investigation of the antihypertensive effect of oral crude stevioside in patients with mild essential hypertension. Phytother. Res..

[36] Reid M., Hammersley R., Hill A.J., Skidmore P. (2007). Long-Term Dietary Compensation for Added Sugar: Effects of Supplementary Sucrose Drinks over a 4-Week Period. Br. J. Nutr..

[37] Njike V.Y., Faridi Z., Shuval K., Dutta S., Kay C.D., West S.G., Kris-Etherton P.M., Katz D.L. (2011). Effects of Sugar-Sweetened and Sugar-Free Cocoa on Endothelial Function in Overweight Adults. Int. J. Cardiol..

[38] Madjd A., Taylor M.A., Delavari A., Malekzadeh R., Macdonald I.A., Farshchi H.R. (2015). Effects on Weight Loss in Adults of Replacing Diet Beverages with Water during a Hypoenergetic Diet: A Randomized, 24-Wk Clinical Trial. Am. J. Clin. Nutr..

[39] Ebbeling C.B., Feldman H.A., Osganian S.K., Chomitz V.R., Ellenbogen S.J., Ludwig D.S. (2006). Effects of Decreasing Sugar-Sweetened Beverage Consumption on Body Weight in Adolescents: A Randomized, Controlled Pilot Study. Pediatrics.

[40] Ebbeling C.B., Feldman H.A., Chomitz V.R., Antonelli T.A., Gortmaker S.L., Osganian S.K., Ludwig D.S. (2012). A Randomized Trial of Sugar-Sweetened Beverages and Adolescent Body Weight. N. Engl. J. Med..

[41] Blackburn G.L., Kanders B.S., Lavin P.T., Keller S.D., Whatley J. (1997). The Effect of Aspartame as Part of a Multidisciplinary Weight-Control Program on Short- and Long-Term Control of Body Weight. Am. J. Clin. Nutr..

[42] Gatenby S.J., Aaron J.I., Jack V.A., Mela D.J. (1997). Extended Use of Foods Modified in Fat and Sugar Content: Nutritional Implications in a Free-Living Female Population. Am. J. Clin. Nutr..

[43] Tordoff M.G., Alleva A.M. (1990). Effect of Drinking Soda Sweetened with Aspartame or High-Fructose Corn Syrup on Food Intake and Body Weight. Am. J. Clin. Nutr..

[44] Raben A., Møller A.C., Vasilaras T.H., Astrup A. (2001). A Randomized 10 Week Trial of Sucrose vs. Artificial Sweeteners on Body Weight and Blood Pressure after 10 Weeks. Obes. Res..

[45] Raben A., Vasilaras T.H., Møller A.C., Astrup A. (2002). Sucrose Compared with Artificial Sweeteners: Different Effects on Ad Libitum Food Intake and Body Weight after 10 Wk of Supplementation in Overweight Subjects. Am. J. Clin. Nutr..

[46] Gostner A., Schäffer V., Theis S., Menzel T., Lührs H., Melcher R., Schauber J., Kudlich T., Dusel G., Dorbath D. (2005). Effects of Isomalt Consumption on Gastrointestinal and Metabolic Parameters in Healthy Volunteers. Br. J. Nutr..

[47] Maki K.C., Curry L.L., Carakostas M.C., Tarka S.M., Reeves M.S., Farmer M.V., McKenney J.M., Toth P.D., Schwartz S.L., Lubin B.C. (2008). The Hemodynamic Effects of Rebaudioside A in Healthy Adults with Normal and Low-Normal Blood Pressure. Food Chem. Toxicol..

[48] Reid M., Hammersley R., Duffy M. (2010). Effects of Sucrose Drinks on Macronutrient Intake, Body Weight, and Mood in Overweight Women over 4 Weeks. Appetite.

[49] Maersk M., Belza A., Stødkilde-jørgensen H., Ringgaard S., Chabanova E., Thomsen H., Pedersen S.B., Astrup A., Richelsen B. (2012). Sucrose-Sweetened Beverages Increase Fat Storage in the Liver, Muscle, and Visceral Fat Depot: A 6-Mo Randomized Intervention Study. Am. J. Clin. Nutr..

[50] Tate D.F., Turner-Mcgrievy G., Lyons E., Stevens J., Erickson K., Polzien K., Diamond M., Wang X., Popkin B. (2012). Replacing Caloric Beverages with Water or Diet Beverages for Weight Loss in Adults: Main Results of the Choose Healthy Options Consciously Everyday (CHOICE) Randomized Clinical Trial. Am. J. Clin. Nutr..

[51] Reid M., Hammersley R., Duffy M., Ballantyne C. (2014). Effects on Obese Women of the Sugar Sucrose Added to the Diet over 28 d: A Quasi-Randomised, Single-Blind, Controlled Trial. Br. J. Nutr..

[52] Peters J.C., Wyatt H.R., Foster G.D., Pan Z., Wojtanowski A.C., Vander Veur S.S., Herring S.J., Brill C., Hill J.O. (2014). The Effects of Water and Non-Nutritive Sweetened Beverages on Weight Loss During a 12-Week Weight Loss Treatment Program. Obesity.

[53] Kuzma J.N., Cromer G., Hagman D.K., Breymeyer K.L., Roth C.L., Foster-Schubert K.E., Holte S.E., Callahan H.S., Weigle D.S., Kratz M. (2015). No Difference in Ad Libitum Energy Intake in Healthy Men and Women Consuming Beverages Sweetened with Fructose, Glucose, or High-Fructose Corn Syrup: A Randomized Trial. Am. J. Clin. Nutr..

[54] Peters J.C., Beck J., Cardel M., Wyatt H.R., Foster G.D., Pan Z., Wojtanowski A.C., Vander Veur S.S., Herring S.J., Brill C. (2016). The Effects of Water and Non-Nutritive Sweetened Beverages on Weight Loss and Weight Maintenance: A Randomized Clinical Trial. Obesity.

[55] Knopp R.H., Brandt K., Arky R.A. (1976). Effects of Aspartame in Young Persons during Weight Reduction. J. Toxicol. Environ. Health.

[56] De Ruyter J.C., Olthof M.R., Seidell J.C., Katan M.B. (2012). A Trial of Sugar-Free or Sugar-Sweetened Beverages and Body Weight in Children. N. Engl. J. Med..

[57] Higgins K.A., Considine R.V., Mattes R.D. (2018). Aspartame Consumption for 12 Weeks Does Not Affect Glycemia, Appetite, or Body Weight of Healthy, Lean Adults in a Randomized Controlled Trial. J. Nutr..

[58] Kinnamon S.C. (2000). A Plethora of Taste Receptors. Neuron.

[59] Running C.A., Craig B.A., Mattes R.D. (2015). Oleogustus: The Unique Taste of Fat. Chem. Senses.

[60] Reed D.R., Tanaka T., McDaniel A.H. (2006). Diverse tastes: Genetics of sweet and bitter perception. Physiol. Behav..

[61] Li X., Staszewski L., Xu H., Durick K., Zoller M., Adler E. (2002). Human receptors for sweet and umami taste. Proc. Natl. Acad. Sci. USA.

[62] Nelson G., Hoon M.A., Chandrashekar J., Zhang Y., Ryba N.J.P., Zuker C.S. (2001). Mammalian Sweet Taste Receptors. Cell.

[63] Ide N., Sato E., Ohta K., Masuda T., Kitabatake N. (2009). Interactions of the Sweet-Tasting Proteins Thaumatin and Lysozyme with the Human Sweet-Taste Receptor. J. Agric. Food Chem..

[64] Behrens M., Meyerhof W. (2011). Gustatory and extragustatory functions of mammalian taste receptors. Physiol. Behav..

[65] Morini G., Bassoli A., Temussi P.A. (2005). From Small Sweeteners to Sweet Proteins: Anatomy of the Binding Sites of the Human T1R2_T1R3 Receptor. J. Med. Chem..

[66] Kim S.-K., Chen Y., Abrol R., Goddard W.A., Guthrie B. (2017). Activation mechanism of the G protein-coupled sweet receptor heterodimer with sweeteners and allosteric agonists. Proc. Natl. Acad. Sci. USA.

[67] Nie Y., Vigues S., Hobbs J.R., Conn G.L., Munger S.D. (2005). Distinct Contributions of T1R2 and T1R3 Taste Receptor Subunits to the Detection of Sweet Stimuli. Curr. Biol..

[68] Meyers B., Brewer M.S. (2008). Sweet Taste in Man: A Review. J. Food Sci..

[69] Liu B., Ha M., Meng X.-Y., Kaur T., Khaleduzzaman M., Zhang Z., Jiang P., Li X., Cui M. (2011). Molecular Mechanism of Species-Dependent Sweet Taste Toward Artificial Sweeteners. J. Neurosci..

[70] Xu H., Staszewski L., Tang H., Adler E., Zoller M., Li X. (2004). Different functional roles of T1R subunits in the heteromeric taste receptors. Proc. Natl. Acad. Sci. USA.

[71] Masuda K., Koizumi A., Nakajima K., Tanaka T., Abe K., Misaka T., Ishiguro M. (2012). Characterization of the Modes of Binding between Human Sweet Taste Receptor and Low-Molecular-Weight Sweet Compounds. PLoS ONE.

[72] Brown R.J., Rother K.I. (2012). Non-Nutritive Sweeteners and their Role in the Gastrointestinal Tract. J. Clin. Endocrinol. Metab..

[73] Striem B.J., Naim M., Lindemann B. (1991). Generation of cyclic AMP in taste buds of the rat circumvallate papilla in response to sucrose. Cell. Physiol. Biochem..

[74] Bernhardt S.J., Naim M., Zehavi U., Lindemann B. (1996). Changes in IP3 and cytosolic Ca^2+^ in response to sugars and non-sugar sweeteners in transduction of sweet taste in the rat. J. Physiol..

[75] Nakagawa Y., Nagasawa M., Mogami H., Lohse M., Ninomiya Y., Kojima I. (2013). Multimodal function of the sweet taste receptor expressed in pancreatic &beta-cells: Generation of diverse patterns of intracellular signals by sweet agonists. Endocr. J..

[76] Ohtsu Y., Nakagawa Y., Nagasawa M., Takeda S., Arakawa H., Kojima I. (2014). Diverse signaling systems activated by the sweet taste receptor in human GLP-1-secreting cells. Mol. Cell. Endocrinol..

[77] Nakagawa Y., Nagasawa M., Yamada S., Hara A., Mogami H., Nikolaev V.O., Lohse M.J., Shigemura N., Ninomiya Y., Kojima I. (2009). Sweet taste receptor expressed in pancreatic beta-cells activates the calcium and cyclic AMP signaling systems and stimulates insulin secretion. PLoS ONE.

[78] Kojima I., Nakagawa Y., Ohtsu Y., Medina A., Nagasawa M. (2014). Sweet Taste-Sensing Receptors Expressed in Pancreatic β-Cells: Sweet Molecules Act as Biased Agonists. Endocrinol. Metab..

[79] Schiffman S.S., Sattely-Miller E.A., Bishay I.E. (2008). Sensory properties of neotame: Comparison with other sweeteners. Sweetness Sweeten..

[80] Moraru C. (2011). Formulating in Sweetness. Prepared Foods.

[81] Gwak M.-J., Chung S.-J., Kim Y.J., Lim C.S. (2012). Relative sweetness and sensory characteristics of bulk and intense sweeteners. Food Sci. Biotechnol..

[82] Kuhn C., Bufe B., Winnig M., Hofmann T., Frank O., Behrens M., Lewtschenko T., Slack J.P., Ward C.D., Meyerhof W. (2004). Bitter taste receptors for saccharin and acesulfame K. J. Neurosci..

[83] Wiet S.G., Beyts P.K. (1992). Sensory characteristics of sucralose and other high intensity sweeteners. J. Food Sci..

[84] Teff K.L., Mattes R.D., Engelman K. (1991). Cephalic phase insulin release in normal weight males: Verification and reliability. Am. J. Physiol.-Endocrinol. Metab..

[85] Just T., Pau H.W., Engel U., Hummel T. (2008). Cephalic phase insulin release in healthy humans after taste stimulation?. Appetite.

[86] Yamazaki M., Sakaguchi T. (1986). Effects of D-glucose anomers on sweetness taste and insulin release in man. Brain Res. Bull..

[87] Hartel B., Graubaum H.-J., Schneider B., Bier A. (1993). The influence of sweetener solutions on the secretion of insulin and the blood glucose level. Eur. Soc. Ecol. Med..

[88] Dhillon J., Lee J.Y., Mattes R.D. (2017). The cephalic phase insulin response to nutritive and low-calorie sweeteners in solid and beverage form. Physiol. Behav..

[89] Smeets P.A.M., de Graaf C., Stafleu A., van Osch M.J.P., van der Grond J. (2005). Functional magnetic resonance imaging of human hypothalamic responses to sweet taste and calories. Am. J. Clin. Nutr..

[90] Bruce D.G., Storlien L.H., Furler S.M., Chisholm D.J. (1987). Cephalic phase metabolic responses in normal weight adults. Metabolism.

[91] Glendinning J.I., Frim Y.G., Hochman A., Lubitz G.S., Basile A.J., Sclafani A. (2017). Glucose elicits cephalic-phase insulin release in mice by activating KATP channels in taste cells. Am. J. Physiol. Regul. Integr. Comp. Physiol..

[92] Yee K.K., Sukumaran S.K., Kotha R., Gilbertson T.A., Margolskee R.F. (2011). Glucose transporters and ATP-gated K^+^ (KATP) metabolic sensors are present in type 1 taste receptor 3 (T1r3)-expressing taste cells. Proc. Natl. Acad. Sci. USA.

[93] Teff K.L., Engelman K. (1996). Oral sensory stimulation improves glucose tolerance in humans: Effects on insulin, C-peptide, and glucagon. Am. J. Physiol. Regul. Integr. Comp. Physiol..

[94] Teff K. (2000). Nutritional implications of the cephalic-phase reflexes: Endocrine responses. Appetite.

[95] Smeets P.A.M., Erkner A., de Graaf C. (2010). Cephalic phase responses and appetite. Nutr. Rev..

[96] Laffitte A., Neiers F., Briand L. (2014). Functional roles of the sweet taste receptor in oral and extraoral tissues. Curr. Opin. Clin. Nutr. Metab. Care.

[97] Ford H.E., Peters V., Martin N.M., Sleeth M.L., Ghatei M.A., Frost G.S., Bloom S.R. (2011). Effects of oral ingestion of sucralose on gut hormone response and appetite in healthy normal-weight subjects. European J. Clin. Nutr..

[98] Brown A.W., Brown M.M., Onken K.L., Beitz D.C. (2011). Short-term consumption of sucralose, a nonnutritive sweetener, is similar to water with regard to select markers of hunger signaling and short-term glucose homeostasis in women. Nutr. Res..

[99] Hall W.L., Millward D.J., Rogers P.J., Morgan L.M. (2003). Physiological mechanisms mediating aspartame-induced satiety. Physiol. Behav..

[100] Ma J., Bellon M., Wishart J.M., Young R., Blackshaw L.A., Jones K.L., Horowitz M., Rayner C.K. (2009). Effect of the artificial sweetener, sucralose, on gastric emptying and incretin hormone release in healthy subjects. Am. J. Physiol.-Gastrointest. Liver Physiol..

[101] Ma J., Chang J., Checklin H.L., Young R.L., Jones K.L., Horowitz M., Rayner C.K. (2010). Effect of the artificial sweetener, sucralose, on small intestinal glucose absorption in healthy human subjects. Br. J. Nutr..

[102] Steinert R.E., Frey F., Töpfer A., Drewe J., Beglinger C. (2011). Effects of carbohydrate sugars and artificial sweeteners on appetite and the secretion of gastrointestinal satiety peptides. Br. J. Nutr..

[103] Temizkan S., Deyneli O., Yasar M., Arpa M., Gunes M., Yazici D., Sirikci O., Haklar G., Imeryuz N., Yavuz D.G. (2014). Sucralose enhances GLP-1 release and lowers blood glucose in the presence of carbohydrate in healthy subjects but not in patients with type 2 diabetes. Eur. J. Clin. Nutr..

[104] Sylvetsky A.C., Rother K.I. (2016). Trends in the consumption of low-calorie sweeteners. Physiol. Behav..

[105] Sylvetsky A.C., Brown R.J., Blau J.E., Walter M., Rother K.I. (2016). Hormonal responses to non-nutritive sweeteners in water and diet soda. Nutr. Metab..

[106] Brown R.J., Walter M., Rother K.I. (2009). Ingestion of Diet Soda Before a Glucose Load Augments Glucagon-Like Peptide-1 Secretion. Diabetes Care.

[107] Brown R.J., Walter M., Rother K.I. (2012). Effects of Diet Soda on Gut Hormones in Youths with Diabetes. Diabetes Care.

[108] Egan J.M., Margolskee R.F. (2008). Taste cells of the gut and gastrointestinal chemosensation. Mol. Interv..

[109] Frank G.K., Kaye W.H., Carter C.S., Brooks S., May C., Fissell K., Stenger V.A. (2003). The evaluation of brain activity in response to taste stimuli—A pilot study and method for central taste activation as assessed by event-related fMRI. J. Neurosci. Methods.

[110] Haase L., Cerf-Ducastel B., Murphy C. (2009). Cortical activation in response to pure taste stimuli during the physiological states of hunger and satiety. NeuroImage.

[111] Frank G.K.W., Oberndorfer T.A., Simmons A.N., Paulus M.P., Fudge J.L., Yang T.T., Kaye W.H. (2008). Sucrose activates human taste pathways differently from artificial sweetener. NeuroImage.

[112] McCutcheon J.E., Beeler J.A., Roitman M.F. (2012). Sucrose-predictive cues evoke greater phasic dopamine release than saccharin-predictive cues. Synapse.

[113] Obermdorfer T.A., Grank G.K.W., Simmons A.N., Wagner A., McCurdy D., Fudge J.L., Yang T.T., Paulus M.P., Kaye W.H. (2013). Altered insula response to sweet taste processing after recovery from anorexia and bulimia nervosa. Am. J. Psychiatry.

[114] Smeets P.A.M., Weijzen P., de Graaf C., Viergever M.A. (2011). Consumption of caloric and non-caloric versions of a soft drink differentially affects brain activation during tasting. NeuroImage.

[115] Chambers E.S., Bridge M.W., Jones D.A. (2009). Carbohydrate sensing in the human mouth: Effects on exercise performance and brain activity. J. Physiol..

[116] Rudenga K.J., Small D.M. (2012). Amygdala response to sucrose consumption is inversely related to artificial sweetener use. Appetite.

[117] Green E., Murphy C. (2012). Altered processing of sweet taste in the brain of diet soda drinkers. Physiol. Behav..

[118] Burke M.V., Small D.M. (2015). Physiological mechanisms by which non-nutritive sweeteners may impact body weight and metabolism. Physiol. Behav..

[119] Ferreira A.V.M., Generoso S.V., Teixeira A.L. (2014). Do low-calorie drinks ‘cheat’ the enteral-brain axis?. Opin. Clin. Nutr. Metab. Care.

[120] Swithers S.E., Ogden S.B., Laboy A.F., Davidson T.L. (2012). Saccharin pre-exposure enhances appetitive flavor learning in pre-weanling rats. Dev. Psychobiol..

[121] Davidson T.L., Martin A.A., Clark K., Swithers S.E. (2011). Intake of high-intensity sweeteners alters the ability of sweet taste to signal caloric consequences: Implications for the learned control of energy and body weight regulation. Q. J. Exp. Psychol..

[122] Griffioen-Roose S., Smeets P.A.M., Weijzen P.L.G., van Rijn I., van den Bosch I., de Graaf C. (2013). Effect of Replacing Sugar with Non-Caloric Sweeteners in Beverages on the Reward Value after Repeated Exposure. PLoS ONE.

[123] Wise R.A. (2005). Forebrain substrates of reward and motivation. J. Comp. Neurol..

[124] Goldfield G.S., Lorello C., Doucet É. (2007). Methylphenidate reduces energy intake and dietary fat intake in adults: A mechanism of reduced reinforcing value of food?. Am. J. Clin. Nutr..

[125] Van de Wouw M., Schellekens H., Dinan T.G., Cryan J.F. (2017). Microbiota-Gut-Brain Axis: Modulator of Host Metabolism and Appetite. J. Nutr..

[126] Alcock J., Maley C.C., Aktipis C.A. (2014). Is eating behavior manipulated by the gastrointestinal microbiota? Evolutionary pressures and potential mechanisms. Bioessays.

[127] Ley R.E. (2010). Obesity and the human microbiome. Curr. Opin. Gastroenterol..

[128] Ley R.E., Turnbaugh P.J., Klein S., Gordon J.I. (2006). Microbial ecology: Human gut microbes associated with obesity. Nature.

[129] Turnbaugh P.J., Ley R.E., Mahowald M.A., Magrini V., Mardis E.R., Gordon J.I. (2006). An obesity-associated gut microbiome with increased capacity for energy harvest. Nature.

[130] David L.A., Maurice C.F., Carmody R.N., Gootenberg D.B., Button J.E., Wolfe B.E., Ling A.V., Devlin A.S., Varma Y., Fischbach M.A. (2014). Diet rapidly and reproducibly alters the human gut microbiome. Nature.

[131] Lobach A.R., Roberts A., Rowland I.R. (2019). Assessing the in vivo data on low/no-calorie sweeteners and the gut microbiota. Food Chem. Toxicol..

[132] Fetissov S.O. (2017). Role of the gut microbiota in host appetite control: Bacterial growth to animal feeding behaviour. Nat. Rev. Endocrinol..

[133] Magnuson B.A., Carakostas M.C., Moore N.H., Poulos S.P., Renwick A.G. (2016). Biological fate of low-calorie sweeteners. Nutr. Rev..

[134] Frankenfeld C.L., Sikaroodi M., Lamb E., Shoemaker S., Gillevet P.M. (2015). High-intensity sweetener consumption and gut microbiome content and predicted gene function in a cross-sectional study of adults in the United States. Ann. Epidemiol..

[135] Palmnas M.S., Cowan T.E., Bomhof M.R., Su J., Reimer R.A., Vogel H.J., Hittel D.S., Shearer J. (2014). Low-dose aspartame consumption differentially affects gut microbiota-host metabolic interactions in the diet-induced obese rat. PLoS ONE.

[136] Uebanso T., Ohnishi A., Kitayama R., Yoshimoto A., Nakahashi M., Shimohata T., Mawatari K., Takahashi A. (2017). Effects of Low-Dose Non-Caloric Sweetener Consumption on Gut Microbiota in Mice. Nutrients.

[137] Suez J., Korem T., Zeevi D., Zilberman-Schapira G., Thaiss C.A., Maza O., Israeli D., Zmora N., Gilad S., Weinberger A. (2014). Artificial sweeteners induce glucose intolerance by altering the gut microbiota. Nature.

[138] Nettleton J.E., Klancic T., Schick A., Choo A.C., Shearer J., Borgland S.L., Chleilat F., Mayengbam S., Reimer R.A. (2019). Low-Dose Stevia (*Rebaudioside* A) Consumption Perturbs Gut Microbiota and the Mesolimbic Dopamine Reward System. Nutrients.

[139] Abou-Donia M.B., El-Masry E.M., Abdel-Rahman A.A., McLendon R.E., Schiffman S.S. (2008). Splenda alters gut microflora and increases intestinal p-glycoprotein and cytochrome p-450 in male rats. J. Toxicol. Environ. Health A.

[140] Brusick D., Borzelleca J.F., Gallo M., Williams G., Kille J., Wallace Hayes A., Xavier Pi-Sunyer F., Williams C., Burks W. (2009). Expert Panel report on a study of Splenda in male rats. Regul. Toxicol. Pharmacol..

[141] Council Spokesperson, Berna Magnuson, Reviews Nature Study on Low-Calorie Sweeteners. https://caloriecontrol.org/council-spokesperson-berna-magnuson-reviews-nature-study-on-low-calorie-sweeteners/.

[142] Nettleton J.E., Reimer R.A., Shearer J. (2016). Reshaping the gut microbiota: Impact of low calorie sweeteners and the link to insulin resistance?. Physiol. Behav..

[143] Bian X., Chi L., Gao B., Tu P., Ru H., Lu K. (2017). The artificial sweetener acesulfame potassium affects the gut microbiome and body weight gain in CD-1 mice. PLoS ONE.

[144] Anderson R.L., Kirkland J.J. (1980). The effect of sodium saccharin in the diet on caecal microflora. Food Cosmet. Toxicol..

[145] Li S., Chen T., Dong S., Xiong Y., Wei H., Xu F. (2014). The Effects of Rebaudioside and on Microbial Diversity in Mouse Intestine. Food Sci. Technol. Res..

[146] Samuel P., Ayoob K.T., Magnuson B.A., Wölwer-Rieck U., Jeppesen P.B., Rogers P.J., Rowland I., Mathews R. (2018). Stevia Leaf to Stevia Sweetener: Exploring Its Science, Benefits, and Future Potential. J. Nutr..

[147] Mattes R.D., Popkin B.M. (2009). Nonnutritive sweetener consumption in humans: Effects on appetite and food intake and their putative mechanisms. Am. J. Clin. Nutr..

[148] Rogers P.J., Carlyle J.A., Hill A.J., Blundell J.E. (1988). Uncoupling sweet taste and calories: Comparison of the effects of glucose and three intense sweeteners on hunger and food intake. Physiol. Behav..

[149] Tordoff M.G., Alleva A.M. (1990). Oral stimulation with aspartame increases hunger. Physiol. Behav..

[150] Yang Q. (2010). Gain weight by “going diet?” Artificial sweeteners and the neurobiology of sugar cravings: Neuroscience 2010. Yale J. Biol. Med..

[151] Ludwig D.S. (2009). Artificially Sweetened Beverages: Cause for Concern. JAMA.

[152] Jamel H.A., Sheiham A., Cowell C.R., Watt R.G. (1996). Taste preference for sweetness in urban and rural populations in Iraq. J. Dent. Res..

[153] Rogers P.J., Blundell J.E. (1989). Separating the actions of sweetness and calories: Effects of saccharin and carbohydrates on hunger and food intake in human subjects. Physiol. Behav..

[154] Mattes R. (1994). Interaction Between the Energy Content and Sensory Properties of Foods.

[155] Anton S.D., Martin C.K., Han H., Coulon S., Cefalu W.T., Geiselman P., Williamson D.A. (2010). Effects of stevia, aspartame, and sucrose on food intake, satiety, and postprandial glucose and insulin levels. Appetite.

[156] Ludwig D.S., Majzoub J.A., Al-Zahrani A., Dallal G.E., Blanco I., Roberts S.B. (1999). High glycemic index foods, overeating, and obesity. Pediatrics.

[157] Reimann F., Habib A.M., Tolhurst G., Parker H.E., Rogers G.J., Gribble F.M. (2008). Glucose sensing in L cells: A primary cell study. Cell Metab..

[158] Jang H.J., Kokrashvili Z., Theodorakis M.J., Carlson O.D., Kim B.J., Zhou J., Kim H.H., Xu X., Chan S.L., Juhaszova M. (2007). Gut-expressed gustducin and taste receptors regulate secretion of glucagon-like peptide-1. Proc. Natl. Acad. Sci. USA.

[159] Margolskee R.F., Dyer J., Kokrashvili Z., Salmon K.S., Ilegems E., Daly K., Maillet E.L., Ninomiya Y., Mosinger B., Shirazi-Beechey S.P. (2007). T1R3 and gustducin in gut sense sugars to regulate expression of Na^+^-glucose cotransporter 1. Proc. Natl. Acad. Sci. USA.

[160] Geraedts M.C.P., Troost F.J., Saris W.H.M. (2011). Different tastants and low-caloric sweeteners induce differential effects on the release of satiety hormones. Food Chem..

[161] Fujita Y., Wideman R.D., Speck M., Asadi A., King D.S., Webber T.D., Haneda M., Kieffer T.J. (2009). Incretin release from gut is acutely enhanced by sugar but not by sweeteners in vivo. Am. J. Physiol.-Endocrinol. Metab..

